# Inhaled steroid/tobacco smoke particle interactions: a new light on steroid resistance

**DOI:** 10.1186/1465-9921-10-48

**Published:** 2009-06-11

**Authors:** Giovanni Invernizzi, Ario Ruprecht, Cinzia De Marco, Roberto Mazza, Gabriele Nicolini, Roberto Boffi

**Affiliations:** 1 Environmental Tobacco Smoke Research Laboratory, Tobacco Control Unit, Fondazione IRCCS Istituto Nazionale dei Tumori/SIMG Italian College GPs, Milan, Italy; 2 Department of Clinical Sciences, University of Parma, Parma, Italy

## Abstract

**Background:**

Inhaled steroid resistance is an obstacle to asthma control in asthmatic smokers. The reasons of this phenomenon are not yet entirely understood. Interaction of drug particles with environmental tobacco smoke (ETS) could change the aerodynamic profile of the drug through the particle coagulation phenomenon. Aim of the present study was to examine whether steroid particles interact with smoke when delivered in the presence of ETS.

**Methods:**

Beclomethasone-hydrofluoralkane (BDP-HFA) pMDI particle profile was studied after a single actuation delivered in ambient air or in the presence of ETS in an experimental chamber using a light scattering Optical Particle Counter capable of measuring the concentrations of particle sized 0.3–1.0, 1.1–2.0, 2.1–3.0, 3.1–4.0, 4.1–5.0, and > 5.1 μm in diameter with a sampling time of one second. The number of drug particles delivered after a single actuation was measured as the difference between total particle number after drug delivery and background particle number. Two groups of experiments were carried out at different ambient background particle concentrations. Two-tail Student's t-test was used for statistical analysis.

**Results:**

When delivered in ambient air, over 90% of BDP-HFA particles were found in the 0.3–1.0 μm size class, while particles sized 1.1–2.0 μm and 2.1–3.0 represented less than 6.6% and 2.8% of total particles, respectively. However, when delivered in the presence of ETS, drug particle profile was modified, with an impressive decrease of 0.3–1.0 μm particles, the most represented particles resulting those sized 1.1–2.0 μm (over 66.6% of total particles), and 2.1–3.0 μm particles accounting up to 31% of total particles.

**Conclusion:**

Our data suggest that particle interaction between inhaled BDP-HFA pMDI and ETS takes place in the first few seconds after drug delivery, with a decrease in smaller particles and a concurrent increase of larger particles. The resulting changes in aerosol particle profile might modify regional drug deposition with potential detriment to drug efficacy, and represent a new element of steroid resistance in smokers. Although the present study does not provide any functional or clinical assessment, it might be useful to advise smokers and non smokers with obstructive lung disease such as asthma or COPD, to avoid to act inhaled drugs in the presence of ETS in order to obtain the best therapeutic effect.

## Introduction

Suspended particles in aerosol phase are subject to dynamic changes, mainly due to the process of coagulation, whereby particles collide with one another, and adhere to form larger particles, due mainly to brownian motion [1,2]. Collisions with bigger particles generate larger particles which work as "scavengers" for smaller particles. The phenomenon is governed by mathematical equations [3-5], and particle coagulation has been observed in experimental exposure chambers [6-8], in indoor settings [3,6,9,10], and in atmospheric environment research [11]. Particle coagulation takes place very rapidly, becoming measurable in a few seconds and continuing up to about a few hours [3-10]. Combustion processes are the main source of primary and secondary submicrometric aerosol particles (particles less than 1.0 μm in diameter) [12]. Environmental tobacco smoke (ETS) is a well studied model of combustion product, being composed by over 4,000 different chemical substances [13-15]. Freshly dispersed ETS is composed mainly by fine particles ranging in diameter 0.02 to 2.0 μm, and displaying a rapid reduction in particle number with a concurrent increase in mean diameter due to particle coagulation during the phase of "aging", the process leading to changes in physical and chemical characterisctics of smoke, which takes place quickly after ETS generation [3,6,9,10].

ETS is one of the most common cause of indoor pollution [13], and is a well recognized worldwide respiratory risk factor [16,17]. Nevertheless, a relevant percentage of asthmatic subjects have been reported to be current smokers (35% of asthmatics presenting to emergengy departments [18], 26% among a series of over 4000 asthma outpatients in a recent survey [19]), and many non-smoker asthmatics are exposed to ETS [20].

Although inhaled steroids are the cornerstone of asthma therapy, their efficacy is dramatically reduced in asthmatic smokers, compromising asthma control, a phenomenon called "steroid resistance" [21-24]. So far, possible interactions between drug particles and ETS have not been evaluated as a possible explanation for inhaled steroid resistance. If the drug is delivered in the presence of ETS, particles might interact with tobacco smoke by modifying the particle frequency as compared to the original pattern with an increase in larger particles. Since drug particle size represents a critical issue for inhaled drug regional deposition, a change in aerodynamic profile could be detrimental to its clinical effect [25].

According to pharmaceutical guidelines, inhaled steroids are studied in ambient air [26], and no concern has yet been risen about the poor air quality in homes polluted by ETS where inhaled drugs are frequently actuated. A special opportunity of particle interaction is represented by the huge concentration of submicrometric (less than 1 μm particles residing in the smoker's lung for up to 3 minutes after the last cigarette puff (the so-called "residual tobacco smoke"), if the inhaled drug is actuated shortly thereafter [27].

The aim of the present report was to investigate if particles size distribution of beclomethasone-hydrofluoralkane pressurized metered dose inhaler (BDP-HFA pMDI) is modified when the pMDI aerosol is delivered in the presence of ETS.

## Materials and methods

**Figure 1:**
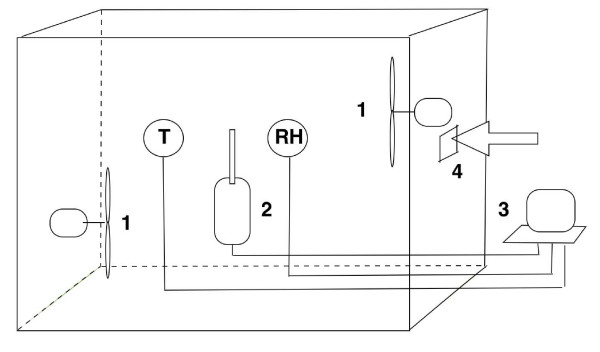
**The figure represents a scheme of the experimental setting**. 1: speed fan. 2: optical particle counter. 3: computer. 4: opening for pMDI drug delivery. T: temperature sensor. RH: relative humidity sensor.

A series of experiments was carried out by measuring BDP-HFA pMDI (Qvar, 3 M Health Care, Ltd, Loughborough, UK) particle size distribution in ambient air (background room air) or in the presence of ETS (background room air additioned with tobacco smoke produced by consuming a smouldering cigarette for 0.5 cm from the tip). The optical particle counter based on real time laser diffraction (model 9012, Metone, Grants Pass, USA) was used, capable of measuring the concentration of particles in the range size of 0.3–1.0, 1.1–2.0, 2.1–3.0, 3.1–4.0, 4.1–5.0, and > 5.1 μm with a sampling time of 1 second [27]. The analyser was placed inside a 6.5 m^3 ^acrylic chamber along with temperature and relative humidity control devices, and with the inner surface coated with antistatic paint to avoid losses of particles due to electrostatic charges. A scheme of the experimental setting is shown in Figure 1. The chamber temperature ranged between 21 and 22°C, while relative humidity beteween 45 and 55%. The study was carried out by delivering a single actuation of BDP-HFA pMDI in two different settings: a) in the presence of ambient air, and b) in the presence of ETS. All data were compensated for coincidence losses, concentration reduction caused by particle sedimentation and adsorption, and for relative humidity interference. Air mixing was provided by two fixed speed fans located inside the box. Chamber air was fully changed after each test. For each experiment, BDP-HFA pMDI particle number was calculated by subtracting the background particle concentration (mean of the last 100 measurements before drug delivery) from the mean of first 100 measurements of total particles number recorded after drug delivery.

Two different groups of experiments were carried out at different times using the same laboratory setting, but with a different serial number of the same particle counter model, and at different ambient background particle concentrations. Background particle concentrations after ETS injection were similar in the different experiments. The first group of experiments was done in triplicate (three ambient and three ETS tests), while the second one was done in quadruplicate. Statistical data analysis was performed with two-tail Student's t-test.

## Results

**Figure 2:**
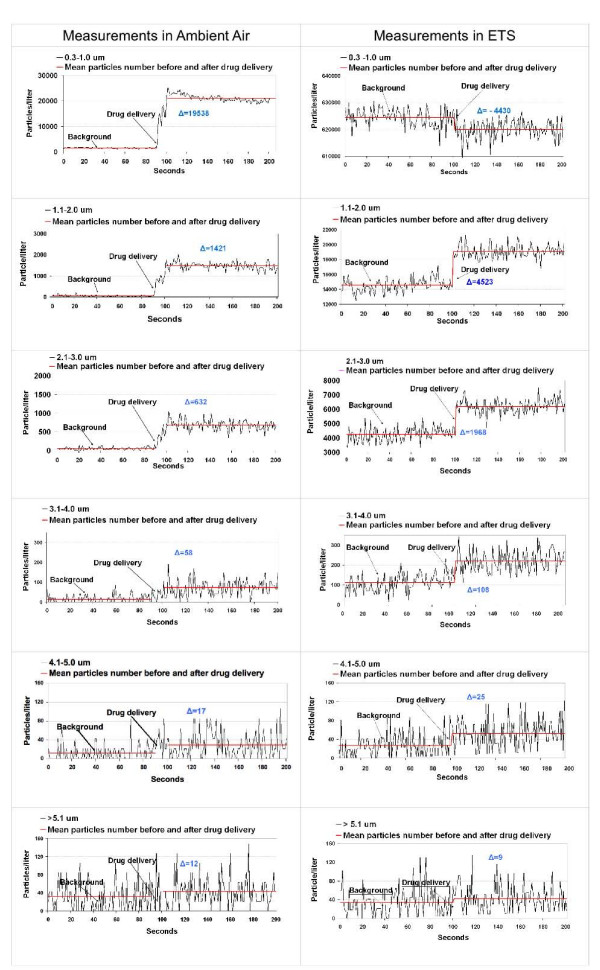
**Particle/liter counts (0.3–1.0 μm through > 5 μm) after a single actuation of BDP-HFA pMDI in ambient air (left) and ETS (right)**. Particle count was measured with a sampling time of one second. Continuous red lines indicate mean particle count of the background and after BDP-HFA pMDI shot. The sign "delta" shows the gradient in particle count after drug delivery(means of 100 counts after delivery less means of 100 counts before delivery). When delivered in ETS, a net decrease in the number of 0.3–1.0 μm particles occurred as compared to delivery in ambient air, while a concurrent increase in the number of larger particles was observed.

An explanatory picture of real time measurement before and after the drug delivery in ambient air is shown in Figure 2, left side, representing the graphical data of test #1, belonging to the first group of experiments (test group 1): immediately after BDP-HFA pMDI actuation the number of particles increased significantly for every particle size (0.3–1.0 μm to > 5.1 μm in diameter) with a prevalence of particles sized 0.3–1.0 μm.

Table 1 shows the details of the data of this group of tests: an increase in the number of particles over the background was observed for every aerodynamic size class after drug delivery, with a mean ± SD increase in p/l after drug delivery of 19277 ± 2292, 1423 ± 249, 593 ± 156, 39 ± 45, 18 ± 32, and 21 ± 43 for particles sized 1.1–2.0, 2.1–3.0, 3.1–4.0, 4.1–5.0, and > 5.1 μm, respectively (pooled data of the 3 tests).

**Table 1:** Test group 1 particle gradient compared to background after a single actuation of BDP-HFA PMDI: decrease in submicrometric and increase in larger particle number in the presence of ETS.

**Particle diameter**	**0.3–1.0 μm**	**1.1–2.0 μm**	**2.1–3.0 μm**	**3.1–4.0 μm**	**4.1–5.0 μm**	**> 5.1 μm**
**Ambient Air**						

Test # 1	19538 ± 2373	1421 ± 251	632 ± 162	58 ± 45	17 ± 35	12 ± 45

Test # 2	19759 ± 1355	1343 ± 191	504 ± 107	21 ± 46	11 ± 30	30 ± 42

Test # 3	18536 ± 849	1505 ± 178	643 ± 128	38 ± 41	25 ± 30	20 ± 42

**Pooled data of 3 tests**	19277 ± 2292	1423 ± 249	593 ± 156	39 ± 45	18 ± 32	21 ± 43

**ETS**						

Test # 4	-4430 ± 4895	4523 ± 1186	1968 ± 668	108 ± 67	25 ± 38	9 ± 40

Test # 5	-3941 ± 5193	4655 ± 1228	2039 ± 741	106 ± 72	24 ± 41	16 ± 49

Test # 6	-3389 ± 5481	4546 ± 9367	2412 ± 4375	103 ± 172	40 ± 52	26 ± 38

**Pooled data of 3 tests**	-3920 ± 5330*	4575 ± 5522*	2140 ± 2584*	106 ± 115*	30 ± 44*	17 ± 43

ETS = Environmental Tobacco Smoke; BDP-HFA pMDI = beclomethasone-hydrofluoralkane pressurized metered dose inhaler; μm = micrometers.

Values are number of particles/liter (mean ± SD).

*P < 0.001 as compared to pooled data in clean air for the same particle diameter.

Background particle concentrations were stable during the tests, with a mean ± SD particle concentration (pooled data) of 1545 ± 163, 51 ± 35, 58 ± 42, 30 ± 34, 13 ± 14 and 35 ± 31 p/l for the same size classes, respectively.

**Figure 3:**
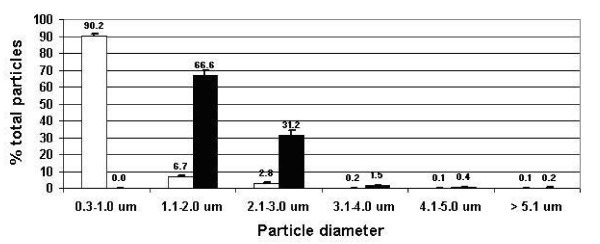
**Tests group 1**. Particle frequency of BDP-HFA pMDI delivered in ambient air (open columns) and in ETS (black columns). Particles sized 0.3–1.0 μm, which predominated in ambient air, were no longer measurable in ETS, particles in the range of 1.0 to 3.0 μm in diameter being mostly represented.

Figure 3 shows the mean ± SD particle frequency of BDP-HFA pMDI: when delivered in ambient air, 90.20 ± 0.97% of the delivered drug was represented by 0.3–1.0 μm particles, while particles 1.1–2.0 and 2.1–3.0 accounted for 6.66 ± 0.54% and 2.78 ± 0.40% of total particles, respectively, with particles over 3.1 μm in diameter accounting for less than 1%.

When the same set of experiments was carried out with BDP-HFA pMDI delivered in the presence of ETS, the distribution of the particle diameter changed, with a significant decrease of particle number for particle sized 0.3–1.0 μm, as compared to background particle concentrations (Fig. 2, right side, representing the graphical data of test #4). By contrast, an excess number of larger particles was recorded as compared to the amount of particles measured in ambient air.

As reported in Table 1, pooled data of the 3 test (#4 – #6) performed in the presence of ETS showed a mean ± SD decrease of -3920 ± 5330 p/l for 0.3–1.0 μm particles, while an increase of 4575 ± 5522, 2140 ± 2584, 106 ± 115, 30 ± 44, and 17 ± 43 was observed for particle sized 1.1–2.0, 2.1–3.0, 3.1–4.0, 4.1–5.0, and > 5.1 μm, respectively. When compared to the tests in ambient air, the number of drug particle delivered in the presence of ETS was statistically significant for all the particle diameters, except for particles over 5.1 μm in size.

ETS particle concentrations were similar in the three different tests, with a mean ± SD particle concentration (pooled data) of 624511 ± 3218, 14542 ± 854, 4239 ± 478, 112 ± 43, 27 ± 24, and 34 ± 28 p/l for particle diameters of 0.3–1.0, 1.1–2.0, 2.1–3.0, 3.1–4.0, 4.1–5.0, and > 5.1 μm, respectively. The exceedingly high prevalence of submicrometric particles with a very low number of coarse particles is consistent with the particle composition of ETS aerosol assessed by previous studies [3,6,9,10].

As shown in Figure 3, the mean ± SD particle frequency of BDP-HFA pMDI delivered in ETS (black bars) was very different as compared to the particle profile of the drug delivered in the presence of ambient air: 0.3–1.0 μm particles disappeared, while particles sized 1.1–2.0 μm and 2.1–3.0 μm predominated with a mean ± SD percentage of 66.62 ± 2.92 and 31.16 ± 2.97. Particles sized 3.1–4.0 μm accounted for a mean ± SD of 1.54 ± 0.09%, while particles 4.1–5.0 and > 5.1 μm in diameter represented less than 1% of total particles.

A similar set of experiments (test group 2), was carried at a different time, with the same model of a particle counter of a different serial number, and with different background air conditions (Table 2). Four test were carried out both for actuations in ambient air and in ETS. When BDP-HFA pMDI was delivered in ambient air, an increase in the number of particles over the background was observed for every aerodynamic size class after drug delivery, with a mean ± SD increase in p/l (pooled data of test #7 – # 10) of 24093 ± 211, 894 ± 22, 89 ± 5, 10 ± 4, 2 ± 2, and 1 ± 1 p/l, for particles sized 1.1–2.0, 2.1–3.0, 3.1–4.0, 4.1–5.0, and > 5.1 μm, respectively.

Background particle concentrations were similar during the experimental session, with a mean ± SD of 94479 ± 1485, 517 ± 105, 68 ± 39, 12 ± 15, 5 ± 10, 4 ± 9, for particles sized 1.1–2.0, 2.1–3.0, 3.1–4.0, 4.1–5.0, and > 5.1 μm, respectively.

**Figure 4:**
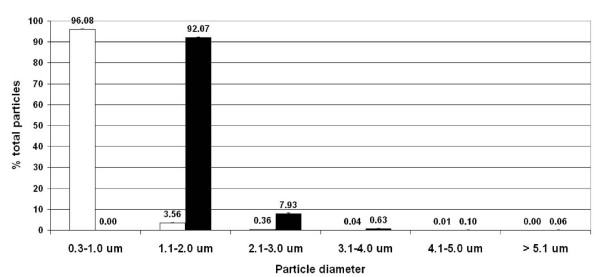
**Tests group 2**. Particles frequency of BDP-HFA pMDI delivered in ambient air (open columns) and in ETS (black columns). Particles sized 0.3–1.0 μm, which predominated in ambient air, were again no longer measurable in ETS, particles in the range of 1.0 to 3.0 μm in diameter being mostly represented.

Figure 4 shows the mean ± SD particle frequency of BDP-HFA pMDI of test group 2 experiments when delivered in ambient air (open bars): the data were similar to test group 1 findings, with a predominance of 0.3–1.0 μm particles which represented 90.20 ± 0.97% of total particles, while particles 1.1–2.0 and 2.1–3.0 accounted for 6.66 ± 0.54% and 2.78 ± 0.40%, respectively, with particles over 3.1 μm in diameter accounting for less than 1%.

The pooled data of tests #11–# 14, carried out in the presence of ETS, showed a net decrease in the number of 0.3–1.0 μm particles (-2412 ± 188), while a mean ± SD increase of 1609 ± 46, 139 ± 9, 11 ± 4, 2 ± 2, 1 ± 2 p/l was observed for particle sized 1.1–2.0, 2.1–3.0, 3.1–4.0, 4.1–5.0, and > 5.1 μm, respectively (Table 2). When compared to the tests in ambient air, the number of drug particle delivered in the presence of ETS was significantly different for particles sized 0.3–1.0, 1.1–2.0, and 2.1–3.0 μm.

**Table 2:** Test group 2 particle gradient compared to background after a single actuation of BDP-HFA PMDI: decrease in submicrometric and increase in larger particle number in the presence of ETS.

**Particle diameter**	**0.3–1.0 μm**	**1.1–2.0 μm**	**2.1–3.0 μm**	**3.1–4.0 μm**	**4.1–5.0 μm**	**> 5.1 μm**
**Ambient Air**						

Test # 7	23419 ± 173	908 ± 18	100 ± 5	9 ± 5	2 ± 1	0 ± 1

Test # 8	25245 ± 210	906 ± 20	80 ± 5	7 ± 2	2 ± 2	1 ± 1

Test # 9	24617 ± 280	903 ± 31	80 ± 6	9 ± 4	2 ± 2	1 ± 1

Test # 10	23092 ± 179	858 ± 19	98 ± 5	13 ± 4	2 ± 2	2 ± 2

**Pooled data of 4 tests**	24093 ± 211	894 ± 22	89 ± 5	10 ± 4	2 ± 2	1 ± 1

**ETS**						

Test # 11	-2633 ± 196	1574 ± 41	140 ± 9	11 ± 3	2 ± 2	0 ± 1

Test # 12	-2623 ± 206	1505 ± 50	136 ± 10	10 ± 3	1 ± 1	1 ± 1

Test # 13	-2180 ± 161	1604 ± 44	127 ± 8	16 ± 5	3 ± 2	3 ± 2

Test# 14	-2212 ± 189	1751 ± 49	151 ± 8	7 ± 4	2 ± 2	1 ± 1

**Pooled data of 4 tests**	-2412 ± 188*	1609 ± 46*	139 ± 9*	11 ± 4	2 ± 2	1 ± 2

ETS = Environmental Tobacco Smoke; BPD-HFA pMDI = beclometasone-hydrofluoroalkane pressurized metered dose inhaler; μm = micrometers.

Values are number of particles/liter (mean ± SD).

*P < 0.001 as compared to pooled data in ambient air for the same particle diameter

ETS particle concentrations were similar in the 4 different tests, with a mean ± SD particle concentration (pooled data) of 584934 ± 2764, 2422 ± 214, 158 ± 58, 25 ± 24, 9 ± 13, and 10 ± 13 p/l, for particle diameters of 0.3–1.0, 1.1–2.0, 2.1–3.0, 3.1–4.0, 4.1–5.0, and > 5.1 μm, respectively.

As shown in Figure 4 (black bars), the mean ± SD particle frequency of BDP-HFA pMDI delivered in ETS changed as compared to the particle profile of the drug delivered in the presence of ambient air: 0.3–1.0 μm particles disappeared, while particles sized 1.1–2.0 μm and 2.1–3.0 μm predominated with a mean (SD) percentage of 92.07 ± 0.1 and 7,93 ± 0.3, respectively, while particles larger that 3.1 μm representing less than 1% of total particles.

## Discussion

Our results indicate that delivery in the presence of ETS can affect pMDI steroid particle size distribution with a shift towards larger particle size taking place in a few seconds. In the two groups of experiments with different ambient backgrounds the particle frequency in ETS showed the disappearance of particles sized 0.3–1.0 μm in diameter, with an increase mainly in 1.1–2.0 and 2.1–3.0 μm particles.

We choose extrafine HFA formulation BDP because it is a widely used inhaled steroid and because it is the only inhaled corticosteroid included in the list of essential drugs of the World Health Organization [28]. Bronchial deposition of drugs depends on particle diameter, the smaller the particles, the easier they reach the most peripheral regions of the lung [29-31]. Due to the extrafine particle profile of BDP-HFA pMDI, the shift towards larger size class distribution did not cause an increase in the percentage of particles sized > 5.1 μm, thus fully preserving the ability of the drug to reach the lung, only particles with diameter < 6.0 μm being considered suitable for this behaviour [29-31].

The growth in particle size might change drug deposition in the small airways, which are involved in inflammation processes and bronchoconstriction both in asthma and in COPD, even though such a principle may not be working for other drugs. In fact, Usmani and co-workers reported that larger particles were more efficacious than smaller particles for the beta-agonist albuterol [30]. Interactions with ETS regard mainly smokers, because of the presence of residual tobacco smoke which can persist in their lung for several minutes aftre the last puff [27], and because of their condition as a category exposed to their own ETS. However, also non smokers are subject to the risk of taking altered inhaled drugs due to ETS, because smoking in the home is still the major cause of exposure to secondhand smoke, especially in children [32].

Although active and passive smoking are regarded as important risk factors for asthma exacerbations, and in spite of the fact that the issue of poor asthma control in current smokers is drawing attention also in the primary care setting [19,33], no special advice to avoid interference of tobacco smoke when using inhaled drugs is reported in asthma guidelines and reccomendations [25,33-39]. Only the paragraph devoted to pentamidine aerosol delivery in the British Society Nebulizer Study Group Report recommends that "patients should not smoke cigarettes for two hours before treatment" [34]. However, if particle interaction can be a problem for asthmatic smokers, it is even more important for patients with chronic obstructive pulmonary disease (COPD), since about 50% of COPD patients on inhaled drug therapy are current smokers [39,40]. Drug prescription leaflets of the most used inhaled drugs do not give any advice to patients regarding neither the timing of actuation in relation to the last puff of cigarette, nor the pollution level of the room where the drugs are delivered.

The results obtained with the real time laser-operated analyser were consistent and repeatable. However this technique has some limitations, such as the possibility of interferences due to coincidence losses in particle count at high particle concentrations: therefore, data for particles sized 0.3–1.0 μm in diameter were corrected according to a proper equation. In addition, our data showed a concurrent increase in the number of particles with larger size class, a result difficult to explain by a technical artifact. Another limitation of the optical particle counter technology is that it measures only particle number and size, without characterizing the kind of particles measured and determining their mass. The results are therefore only suggestive of particle coagulation. Further studies with different methodologies such as Andersen cascade impactor and Scanning Electron Microscopy technology are needed to provide such an information and confirm the presence of the phenomenon [26]. As for temperature and relative humidity, ETS addition to the chamber did not alter these parameters as compared to ambient air experiments, probably because of the very limited amount of smoke inflated into the test chamber (0.5 cm of a smouldering cigarette). Other physical processes affecting aerosol dynamics, like charge effects of ETS particles and hygroscopic properties of inhaled drug particles could play a role in the observed findings, and deserve suitable research [2].

BDP-HFA pMDI particle profile showed small differences in the two different tests groups carried out at different times in ambient air: particle frequency was 90.2%, 6.7%, and 2.8% in test group 1, and 96.2%, 3.5%, and 0.4% in test group 2, for particles sized 0.3–1.0, 1.1–2.0, and 2.1–3.0 μm in diameter, respectively (see Figure 3 and 4). The differences were more relevant for drug delivery in ETS, with a particle frequency of 66.6%, 31.2%, and 1.5%, and of 92.2%, 7.9% and 0.63% for particles sized 1.1–2.0, 2.1–3.0, and 3.1–4.0 μm in diameter, in two test groups, respectively. Such discrepancies could be explained because two different analyzers were used, and due to the different ambient air background.

It should be underlined that, although in the presence of ETS the aerodynamic profile of BDP-HFA pMDI particles was deeply altered, the "load" of particles ranging between 0.3 and 3.0 μm in diameter, altogether, did not change when comparing ambient air *vs *ETS profile, accounting for over 97% of total particles, suggesting only minor changes in overall regional deposition of the drug [29-31].

Although much higher than the ambient air counterpart, the mean levels of background submicrometric particle concentrations used in the experiments with ETS represent real world indoor concentrations which can be currently encountered in ETS polluted places, reaching levels of several hundred thousand particles per liter [2,3,9,10,27].

The possibility of spontaneous "self coagulation" of ETS deserve a comment. A particle size increase between 20 and 50% along with a concurrent reduction in total particle number were reported by Morawska and co-workers during the first 30 to 60 minutes after of ETS production [6], while Ning et al. showed a drop from ~1 × 10^5 ^to ~6 × 10^4^/cm^3 ^in total ETS particles in the first 15 minutes since generation [10]. In our experiments no detectable change in particle number was observed in ETS background during the 100 seconds preceding drug delivery, thus excluding such a phenomenon to explain the rapid drop in submicrometric particles we observed just after the pMDI actuations.

Another issue to be discussed is the rapidity of particle interaction. In a recent study, Seipenbush and co-workers were able to demonstrate that the injection of 0.2 μm sebacate droplets into a background aerosol of platinum nanoparticles induced a heterogeneous coagulation process measurable in the first 4 minutes of interaction [8]. The authors carried out chemical and morphological analysis of collected particles, and concluded that nanoparticles coagulate very rapidly after the injection of sebacate particles, in an experimental setting which is very similar to ours.

## Conclusion

In summary, our data showed that the particle profile of the inhaled steroid BDP-HFA pMDI is altered when the drug is delivered in the presence of ETS. Although further studies are needed to confirm these findings, and although the present study does not provide any functional or clinical assessment, the results are relevant to the phenomenon of steroid resistance [22].

Smoking has been shown to impair the clinical response to systemic steroids through biochemical mechanisms affecting steroid pharmacodynamics at the cellular level [41,42]. Our data suggest that particle interaction and growth should be taken into account as another mechanism contributing to the reduced clinical efficacy of inhaled steroid medications in smokers, adding a new piece of evidence. In view of the present findings, it seems reasonable that smokers with obstructive lung disease such as asthma or COPD should be advised, in addition to stop smoking, to act their inhaled drugs in ETS-free ambient air, and far from the last cigarette puff, in order to obtain the best therapeutical effect. Likewise, also non-smokers should receive instructions about the influence of indoor ETS pollution on their inhaled medications.

## Competing interests

The authors declare that they have no competing interests.

## Authors' contributions

GI and AR conceived the study, carried out the particle interaction studies, and performed the statistical analysis. CDM, RM, GN and RB participated in the design of the study, and helped to draft the manuscript. All authors read and approved the final manuscript.
